# Amino acid substitutions V63I or A37S/I61T/V63I/V100A in the PA N-terminal domain increase the virulence of H7N7 influenza A virus

**DOI:** 10.1038/srep37800

**Published:** 2016-11-25

**Authors:** Meng Hu, Hin Chu, Ke Zhang, Kailash Singh, Cun Li, Shuofeng Yuan, Billy K. C. Chow, Wenjun Song, Jie Zhou, Bo-Jian Zheng

**Affiliations:** 1 Department of Microbiology, Li Ka Shing Faculty of Medicine, The University of Hong Kong, Hong Kong SAR, China; 2 School of Biological Sciences, Faculty of Science, The University of Hong Kong, Hong Kong SAR, China

## Abstract

The PA N-terminal domain (PA-Nter) is essential for viral transcription and replication. Here we identified PA-Nter substitutions A37S, I61T, V63I and V100A in recently emerged avian influenza A viruses (IAVs) with potential effect on virus pathogenicity and/or host adaptation. We introduced the identified PA-Nter substitutions into avian H7N7 IAV by reverse genetics. Our results showed that single substitution V63I and combined substitutions, I61T/V63I and A37S/I61T/V63I/V100A (Mfour), significantly increased virus growth capacity in mammalian cells. Meanwhile, these substitutions conferred higher virus transcription/replication capacity by producing more mRNA, cRNA and vRNA. Consistently, the polymerase activity and the endonuclease activity were enhanced by these PA-Nter substitutions. Notably, substitutions V63I and Mfour strongly increased virus replication and virulence in mice. Collectively, our findings demonstrated that the PA-Nter substitutions V63I and Mfour enhanced IAV pathogenicity through modification of the polymerase activity and the endonuclease activity, which added to the evolving knowledge of IAV virulence determinants.

In the past decades, more than ten different subtypes of avian influenza A viruses (IAVs) have overcome species barriers and transmitted to humans, posing great threats to the public health^1^. According to WHO reports, avian H5N1 IAV has caused 846 cases of human infections with 449 deaths since 2003^2^. Avian H7N7 IAV caused 89 cases of human infections with one death in the Netherlands in 2003^3^. Apart from these infection cases, at least 1,000 individuals were exposed to the avian H7N7 IAV during the outbreaks in the Netherlands and abroad during 2003 and 2004^4^. Additionally, the avian H7N7 IAV caused three other cases of human infections in Italy in 2013^5^. Moreover, several novel avian IAVs have emerged and infected humans in the past three years, including H7N9^6^, H5N6^7^ and H10N8 IAVs^8^,^9^. The recurrence of avian IAVs together with ongoing emergence of novel avian IAVs underscore the need for a better understanding on avian IAV pathogenicity.

The pathogenicity of avian IAVs are polygenic, being determined in part by the constellation of virus genes^10^. PA is a component of the IAV RNA polymerase complex that is essential for viral transcription and replication^1^. The N-terminal region of PA (PA-Nter, 1–197 amino acids) is a major functional domain that possesses protease activity^11^ and promoter binding activity^12^,^13^. Moreover, the PA-Nter domain functions as an endonuclease that snatches capped primers from host mRNAs for the initiation of virus mRNA transcription^14^,^15^. Hara *et al*. found PA-Nter substitutions D108A and K134A completely blocked the endonuclease activity^11^. Crepin *et al*. also confirmed a number of PA-Nter mutations (E80A, R84A, D108A, E119A, Y130A, K134A and K137A) that inhibited the endonuclease activity^16^. Additionally, recent studies have demonstrated that PA-Nter substitutions may contribute to the high pathogenic phenotype of avian IAVs^17^,^18^,^19^,^20^,^21^, though the underlying molecular mechanisms are not completely understood. For example, the PA-Nter substitutions T85I and G186S have been reported to enhance the polymerase activity of A(H1N1)pdm09 IAV *in vitro*^17^. The PA-Nter T97I substitution increases the virulence of H5N2 IAV^18^ and contributes to mammalian adaptation of H5N1 IAV in mice^19^. The PA-Nter K142E has been shown to increase the pathogenicity of H5N1 IAV *in vivo* when combined with mutation PB2-E627K^20^,^21^. Together, identification of virulence determinants in PA-Nter and the underlying mechanisms are required in order to fully understand the IAV pathogenicity.

In this study, we identified PA-Nter substitutions A37S, I61T, V63I and V100A in recently emerged avian IAVs with potential effect on virus pathogenicity and/or host adaptation by comprehensive bioinformatics analyses. To investigate the biological significance of these PA-Nter substitutions, we selectively generated mutant viruses of A/Netherlands/219/2003 (H7N7) harboring single or combined substitutions, I61T, V63I, I61T/V63I, A37S/V100A and A37S/I61T/V63I/V100A (Mfour) by reverse genetics. Virus growth capacity, transcription/replication capacity, the polymerase activity and the endonuclease activity of these mutants were compared with those of the wild type *in vitro*. We further investigated the effect of the substitutions V63I and Mfour on virus replication and virulence in mice. Notably, we demonstrated that the PA-Nter substitutions, V63I or Mfour, conferred enhanced virus pathogenicity *in vitro* and *in vivo*. Our study may help to understand the pathogenicity of the emerging avian IAVs and other IAVs carrying these substitutions, which adds to the evolving knowledge of IAV virulence determinants and facilitates the assessment of pandemic risks.

## Results

### Identification of PA-Nter substitutions that may enhance virus pathogenicity and/or host adaptation

By sequence alignment and analyses, we identified amino acid variations at positions 37, 61, 63 and 100 of PA-Nter among influenza A viruses (IAVs) from human infections (Table 1). We found that most IAV subtypes presented 37 alanine (37A), 61 isoleucine (61I), 63 valine (63V) and 100 valine (100V) in PA-Nter. However, these residues were substituted to be 37 serine (37S), 61 threonine (61T) and 63 isoleucine (63I) in almost all the recently emerged avian IAVs, including H7N9^6^,^22^, H5N6 in 2015^7^,^23^, H10N8^8^,^9^ and H9N2 since 2014^24^ (Table 1). Moreover, the majority of H7N9 and H5N6 IAVs isolated in 2015 possessed alanine (A) at position 100. Thus, our data suggested that the PA-Nter substitutions A37S, I61T, V63I and V100A, singly or in combination, might confer avian IAV growth and/or adaptation advantages in humans. Moreover, we examined PA-Nter sequences of human isolates of avian H7N9 and avian H7N7, which showed high phylogenetic similarity with each other^6^. Our analysis showed that the PA-Nter of all the human H7N7 isolates harbored 37A/61I/63V/100V while ~90% of H7N9 isolates presented 37S/61T/63I/100A (Table 1). Further sequence inspection revealed that avian H7N7 IAV with PA-Nter 37S, 61T, 63I or 100A had already been detected in mammalian or avian species (Supplementary Table S1). In addition, the percentages of avian H7N7 isolates bearing these PA-Nter substitutions increased in avian hosts since 2013 (Supplementary Table S1). On the basis of these findings, we speculated the PA-Nter substitutions at positions 37, 61, 63 and 100 might enhance IAV replication and pathogenicity, which could promote virus transmission to humans. Therefore, we were obliged to identify the biological significance of these PA-Nter substitutions in the background of avian H7N7 IAV.

### Effect of substitutions on virus growth

**Figure 1:**
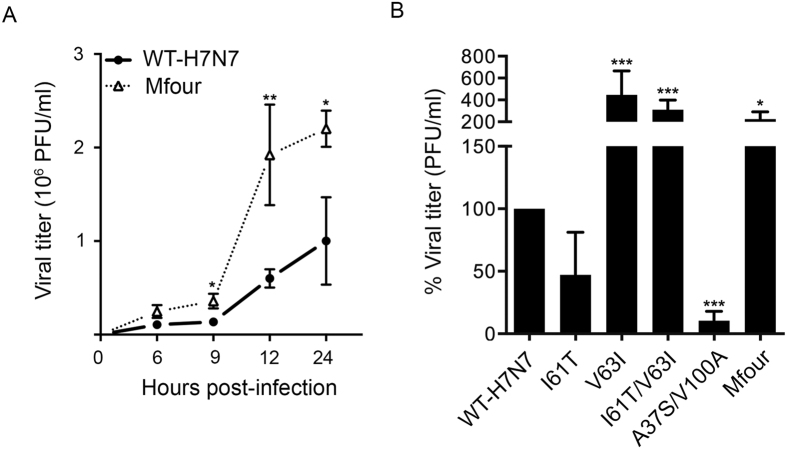
Comparison of virus growth in MDCK cells. (**A**) MDCK cells were infected with WT-H7N7 and A37S/I61T/V63I/V100A (Mfour) viruses at a multiplicity of infection (MOI) of 1. Cell culture supernatants were collected at 6, 9, 12 and 24 hours post-infection (hpi) for virus titration in MDCK cells by plaque assay. Results shown are means ± standard deviation (SD) of independent experiments performed at least three times. * and ** represent *P* < 0.05 and *P* < 0.01, respectively, according to two-tailed Student’s *t*-test compared to that of WT-H7N7. (**B**) MDCK cells were infected with WT-H7N7 and mutant viruses I61T, V63I, I61T/V63I, A37S/V100A and Mfour at an MOI of 1. Cell culture supernatants were collected at 8 hpi for virus titration. Experiments were independently performed at least three times. Data presented are the percentages of viral titers of the indicated mutant viruses relative to that of WT-H7N7 (means ± SD). *** represents *P* < 0.001, according to a one-way ANOVA followed by a Dunnett’s test.

As PA-Nter residues of H7N9 IAV differed from those of WT-H7N7 exclusively at positions 37, 61, 63 and 100, we first asked whether these four substitutions affected virus growth capacity. The plasmid-based reverse genetics of avian H7N7 IAV was established (Supplementary Fig. S1). Recombinant viruses WT-H7N7 and A37S/I61T/V63I/V100A (Mfour) were generated by reverse genetics (Supplementary Figs S2 and S3). To compare the growth capacity of the WT-H7N7 virus and the Mfour mutant virus, we inoculated the viruses into MDCK cells at a multiplicity of infection (MOI) of 1 and harvested supernatants at the indicated time points. As shown in Fig. 1A, viral titers of Mfour were 2.6-fold (*P* = 0.0159), 3.2-fold (*P* = 0.0025) and 2.2-fold (*P* = 0.0286) higher than those of WT-H7N7 at 9, 12 and 24 hours post-infection (hpi), respectively. Therefore, the substitution Mfour (A37S/I61T/V63I/V100A) enhanced virus growth of avian H7N7 IAV in mammalian cells.

Next, we generated additional mutant viruses to delineate the effect of PA-Nter substitutions. Recombinant viruses carrying the single substitutions I61T and V63I were obtained by reverse genetics (Supplementary Figs S2 and S3). As the effect of 37A and 100V on virus pathogenicity had been previously reported in the background of H7N9 IAV^25^, we did not investigate these two substitutions individually in this study. Considering the possibility that the PA-Nter substitutions might occur simultaneously, as shown in several IAV isolates in nature (Table 1 and Supplementary Table S1), we generated recombinant viruses bearing combined substitutions, including I61T/V63I and A37S/V100A (Supplementary Figs S2 and S3).

To evaluate the impact of the substitutions on virus growth, we inoculated recombinant viruses into MDCK cells at an MOI of 1 and harvested supernatants at 8 hpi. As shown in Fig. 1B, titers of the mutant viruses V63I, I61T/V63I and Mfour were substantially higher than that of WT-H7N7 (*P* < 0.05), whereas the titer of the A37S/V100A mutant virus was lower than that of WT-H7N7 (*P* < 0.001). At the same time, the titer of the I61T mutant virus was not statistically different from that of WT-H7N7. Thus, our data indicated that V63I might be able to override the effect of suppressive residue(s) and play a predominant role in determining the virus growth capacity. Collectively, the PA-Nter substitutions V63I, I61T/V63I and Mfour (A37S/I61T/V63I/V100A) increased virus growth capacity in mammalian cells, whereas the substitution A37S/V100A posed a decreased effect.

### Effect of substitutions on the synthesis of viral mRNA, cRNA and vRNA

**Figure 2:**
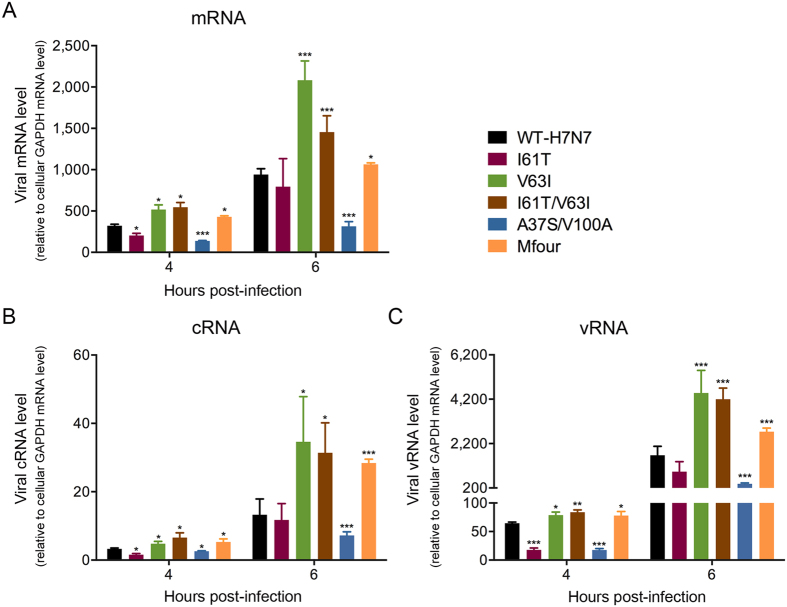
Quantification of viral mRNA, cRNA and vRNA. MDCK cells were infected by WT-H7N7 and mutant viruses I61T, V63I, I61T/V63I, A37S/V100A and Mfour at an MOI of 2. Cell lysates were harvested at 4 and 6 hpi. Viral mRNA, cRNA and vRNA were extracted and quantified. Experiments were independently performed three times. Viral mRNA, cRNA and vRNA were quantified and normalized with that of cellular GAPDH mRNA (means ± SD).

Virus mRNA, cRNA and vRNA were synthesized during viral transcription and replication processes^26^. The PA-Nter plays essential roles in viral transcription and replication^11^,^12^,^13^,^14^. To further investigate the effect of the PA-Nter substitutions I61T, V63I, I61T/V63I, A37S/V100A and Mfour (A37S/I61T/V63I/V100A) on viral transcription and replication, we utilized the abundance of virus mRNA, cRNA and vRNA as indicators. The viruses were inoculated into MDCK cells at an MOI of 2 and cell lysates were harvested at 4 and 6 hpi. Our results showed that the V63I, I61T/V63I and Mfour mutant viruses produced significantly higher levels of mRNA (Fig. 2A), cRNA (Fig. 2B) and vRNA (Fig. 2C) than those of WT-H7N7 at 4 and 6 hpi (*P* < 0.05). Among these substitutions, V63I resulted in the highest amount of mRNA, cRNA and vRNA at 6 hpi (Fig. 2). In contrast, the A37S/V100A mutant virus produced a significantly lower amount of mRNA, cRNA and vRNA than WT-H7N7 at 4 and 6 hpi (*P* < 0.05) (Fig. 2). The I61T mutant virus produced slightly reduced levels of mRNA (*P* = 0.0120), cRNA (*P* = 0.0378) and vRNA (*P* < 0.001) when compared with those of WT-H7N7 at 4 hpi (Fig. 2). Taken together, these results suggested that V63I alone and the combined substitutions I61T/V63I and Mfour (A37S/I61T/V63I/V100A) increased viral transcription and replication by producing more mRNA, cRNA and vRNA in mammalian cells.

### Effect of substitutions on the polymerase activity

**Figure 3:**
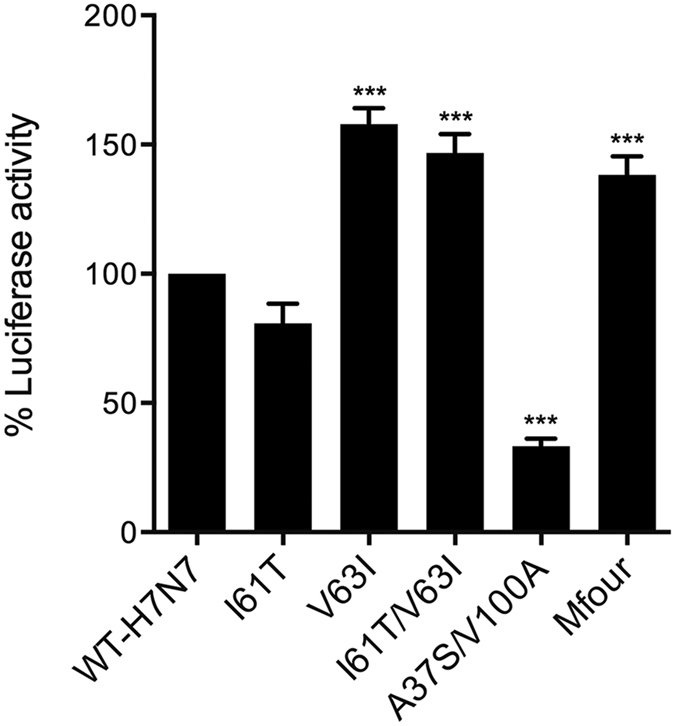
Comparison of the polymerase activity in 293-T cells. 293-T cells were transfected with the plasmids pHW2000-PB1, -PB2, -NP and -PA (with and without the substitutions), together with a plasmid encoding firefly luciferase reporter flanked by IAV untranslated region as well as a plasmid encoding eGFP as an internal control. Luciferase activity was determined at 24 hours post transfection. Experiments were independently performed three times. Data presented are the relative polymerase activity of the indicated mutants to that of WT-H7N7 (means ± SD).

The polymerase complex, composed of PA, PB1 and PB2, together with NP, are responsible for viral transcription and replication^26^. Minireplicon assays were performed to evaluate the impact of the PA-Nter substitutions I61T, V63I, I61T/V63I, A37S/V100A and Mfour (A37S/I61T/V63I/V100A) on the polymerase activity in human 293-T cells. Our data showed that the polymerase complex carrying V63I and the combined substitutions I61T/V63I and Mfour significantly increased the polymerase activity compared to that of WT-H7N7 (*P* < 0.001) (Fig. 3). In contrast, A37S/V100A substantially decreased the polymerase activity when compared to that of WT-H7N7 (*P* < 0.001) (Fig. 3). Hence, the PA-Nter substitutions V63I alone and the combined substitutions (I61T/V63I and Mfour) imposed higher polymerase activity in human cells.

### Effect of substitutions on the endonuclease activity

**Figure 4:**
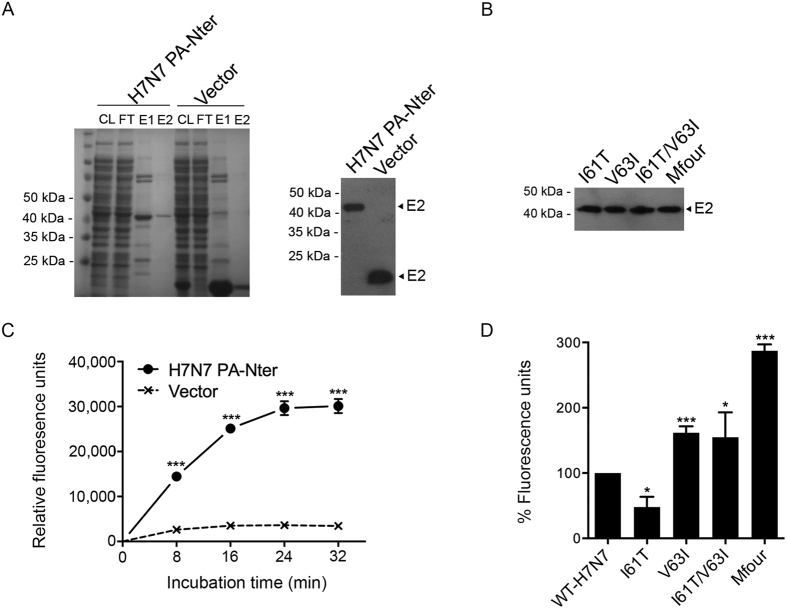
Comparison of the endonuclease activity of virus PA-Nter proteins expressed in *E. coli*. Virus PA-Nter proteins were expressed in *E. coli*, purified, and identified by SDS-PAGE and Western blot using Anti-His antibodies. After concentrated and quantified, the PA-Nter proteins were then incubated with DNA substrates, which contained FAM and BHQ conjugated to the 5′ and 3′ ends, respectively. Fluorescence signals were released and measured at the indicated time points. The purified protein from unmodified vector was achieved and used as a negative control. (**A**) The Coomassie-stained SDS-PAGE image and Western blot result of WT-H7N7 PA-Nter and negative control protein (Vector). CL, cell lysates; FT, flow-through (wash fractions); E1, 1st eluates; and E2, 2nd eluates. E2 proteins were concentrated and used for the endonuclease assay. (**B**) Western blot result of purified PA-Nter proteins of the indicated mutants. (**C**) Endonuclease assay of the WT-H7N7 PA-Nter protein. Data represented are means ± SD of independent experiments performed at least three times. (**D**) Comparison of the endonuclease activity of the PA-Nter proteins of WT-H7N7 and the indicated mutants. Fluorescence signals were collected at 32 minutes after incubation. The experiments were independently performed at least three times. Results presented are the relative endonuclease activity of PA-Nter proteins of the indicated mutants to that of WT-H7N7 (means ± SD).

The PA-Nter functions as an endonuclease that cleaves 10–13 nucleotides from host pre-mRNAs, thereby snatching primers for virus mRNA transcription^14^,^15^. To investigate whether the PA-Nter substitutions I61T, V63I, I61T/V63I, A37S/V100A and Mfour (A37S/I61T/V63I/V100A) affect the endonuclease activity, we cloned the virus PA-Nters (with and without these substitutions) into pET-32a (+) expression vector (Supplementary Fig. S4) and expressed PA-Nter proteins in *E. coli*. Purified PA-Nter proteins were obtained in the second eluates (E2) (Fig. 4A and 4B) and used for the subsequent fluorescence-based endonuclease assays. As shown in Fig. 4C, the WT-H7N7 PA-Nter protein retained the endonuclease activity when expressed in *E. coli*. We then compared the endonuclease activity of PA-Nter proteins of WT-H7N7 and mutants. The result showed that the PA-Nter proteins of the substitutions V63I, I61T/V63I and Mfour exhibited significantly higher endonuclease activity than that of WT-H7N7 (*P* < 0.05), whereas the PA-Nter I61T protein showed lower endonuclease activity than that of WT-H7N7 (*P* = 0.0119) when compared to that of WT-H7N7 (Fig. 4D). Thus, the substitution V63I alone and combined substitutions I61T/V63I and Mfour, significantly enhanced the PA-Nter endonuclease activity, thereby increasing viral transcription and subsequent replication.

### Effect of substitutions V63I and Mfour (A37S/I61T/V63I/V100A) on viral pathogenicity in mice

**Figure 5:**
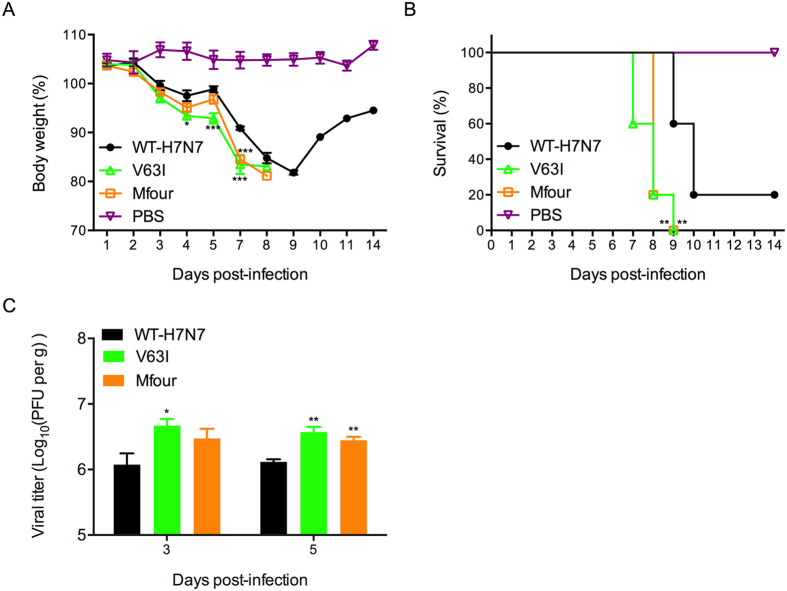
Comparison of viral pathogenicity of WT-H7N7 and mutant viruses V63I and Mfour in mice. Six to eight-week-old BLAB/c mice were intranasally inoculated with 120 PFU of the indicated viruses or PBS. (**A**) Mouse body weight was monitored daily for up to 14 days. The mice that lost more than 20% of their initial weight were humanely euthanized. Data shown represents the average body weight changes (means ± SD). (**B**) Survival rates of the mice were also recorded. ** represented *P* < 0.01, according to the Log-rank test when compared to that of WT-H7N7 virus. (**C**) Three mice in the indicated groups were euthanized and lung tissues were excised on days 3 and 5 post-infection. The lung virus titers were determined by plaque assay (means ± SD).

As demonstrated above, the PA-Nter substitution Mfour (A37S/I61T/V63I/V100A) was prevalent in the majority of recently emerged IAVs, which increased the virus growth and transcription/replication capacity *in vitro*. At the same time, V63I was indicated to pose a dominant effect on enhancing the virulence among the tested substitutions. Thus, we next selectively investigated the effect of these two substitutions on virus pathogenicity *in vivo*. BALB/c mice were intranasally inoculated with WT-H7N7, V63I mutant virus, Mfour mutant virus or PBS. As shown in Fig. 5A, mice infected with the V63I and Mfour mutant viruses showed significantly more severe body weight loss than that of WT-H7N7 infected mice. Correspondingly, the survival rate of V63I and Mfour mice were significantly lower than that of WT-H7N7 mice (*P* < 0.01). All the V63I and Mfour mice succumbed to the infection by day 9 while 20% of the WT-H7N7 mice survived the infection (Fig. 5B). Moreover, the V63I mice showed higher viral titers in lung tissues on days 3 and 5 post-infection (*P* < 0.05), while the Mfour exhibited significantly higher lung viral titers on day 5 post-infection (*P* = 0.0065) (Fig. 5C). Thus, we demonstrated that the PA-Nter substitutions V63I and Mfour enhanced virus growth and pathogenicity *in vivo*.

### Effect of substitutions on the PA-Nter structure

**Figure 6:**
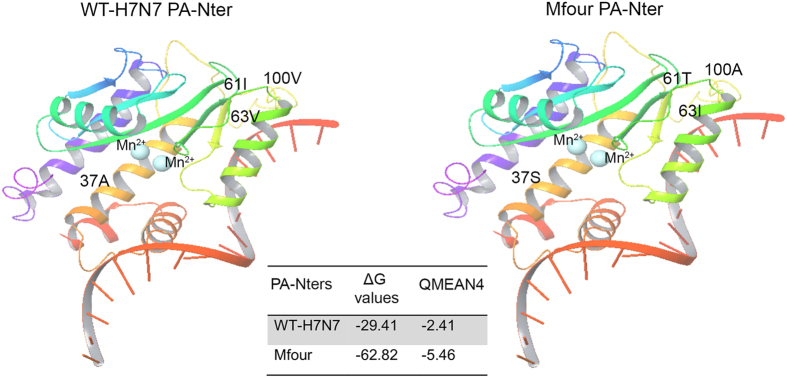
Comparison of the PA-Nter models of WT-H7N7 and Mfour. The 3D PA-Nter models of WT-H7N7 and Mfour were generated and docked with Mn^2+^ ions (indicated by light blue ball) and an ssDNA substrate, respectively. ΔG values were calculated to obtain affinity of the PA-Nter models of WT-H7N7 and Mfour towards the ligand molecules. Differences in WT-H7N7 and Mfour PA-Nter models were aligned with a QMEAN 4 and scored.

The 3D models of WT-H7N7 and Mfour (A37S/I61T/V63I/V100A) PA-Nters were generated and validated. Molecular docking was performed to identify the potential differences between the PA-Nter structures of WT-H7N7 and Mfour. As shown in Fig. 6, the ΔG value of Mfour was substantially lower than that of WT-H7N7, suggesting an increased affinity of the PA-Nter towards the same ligand molecule. The 3D structural differences of WT-H7N7 and Mfour PA-Nter models were showed in Fig. 6 and Supplementary Table S2. Interestingly, the Mfour PA-Nter model used a different table (3hw6) with a QMEAN 4 of −5.46, whereas the WT-H7N7 PA-Nter model best aligned with 3hw4 with a QMEAN 4 of −2.41 (Supplementary Table S2). Collectively, the PA-Nter model of Mfour showed substantial differences from those of WT-H7N7 in both 3D structure and affinity towards the ligand molecule.

## Discussion

In this study, we identified PA-Nter substitutions at positions 37, 61, 63 and 100 in the recently emerged avian influenza A viruses (IAVs) that played key roles in promoting virus replication and might facilitate virus transmission to human hosts. We found that the PA-Nter substitution V63I alone and combined substitutions, I61T/V63I and Mfour (A37S/I61T/V63I/V100A), enhanced virus growth capacity when compared to that of wild type avian H7N7 IAV in mammalian cells. At the same time, these substitutions potentiated viral transcription and replication by producing more virus mRNA, cRNA and vRNA in infected cells. Furthermore, the polymerase activity and the endonuclease activity were augmented by these PA-Nter substitutions. Notably, the V63I and Mfour substitutions enhanced virus growth and virulence in mice. Collectively, our data demonstrated that the substitution V63I alone and the combined substitution Mfour (A37S/I61T/V63I/V100A) increased viral pathogenicity of avian H7N7 IAV through modification of the polymerase activity and the endonuclease activity.

Genetic determinants in virus genes (i.e. HA^27^, NA^28^, PB2^29^, PB1-F2^30^, NEP^31^ and NS^32^) have been reported to contribute to increased pathogenicity and/or adaptation in human or mammalian hosts. Despite these extensive studies, the molecular determinants for IAV virulence and cross-species adaptation in humans remain incompletely understood^33^. Previous studies have identified substitutions in PA enhanced the virulence of IAVs^18^,^34^,^35^,^36^,^37^. Interestingly, the majority of PA virulence determinants were found to cluster in PA-Nter, suggesting a critical role of PA-Nter in IAV pathogenicity. In this study we characterized the effect of PA-Nter substitutions I61T, V63I, I61T/V63I, A37S/V100A and Mfour (A37S/I61T/V63I/V100A) on virus pathogenicity in the context of avian H7N7 IAV. Importantly, the Mfour and V63I substitutions were identified as virulence determinants, which added to the evolving knowledge of IAV pathogenicity and benefited the surveillance of IAVs.

We identified PA-Nter substitutions at positions 37, 61, 63 and 100 in recently emerged IAVs, including the majority of H7N9 and H5N6 IAVs isolated in 2015. Avian H7N9 IAV has caused a large number of severe human infections in China since 2013^6^. The virus is a reassortant with six internal genes derived from H9N2 IAV^38^. Interestingly, we found that the majority of avian H7N9 IAV presented PA-Nter 37S/61T/63I/100A whereas avian H9N2 IAV isolated before 2014 harbored 37A/61I/63V/100V. As H7N9 IAV is considerably more pathogenic than avian H9N2 IAV in humans^22^,^39^, the substitution A37S/I61T/V63I/V100A may be one of the contributing factors that account for the higher pathogenicity. Note that human H9N2 isolates during 2014 and 2015 present the PA-Nter substitution 37S/61T/63I/100V, which may suggest that the H9N2 IAV is continuously evolving and posing a pandemic potential to humans.

Virus mRNA transcription is primer-dependent, which requires caps snatched from the host cells in the initiation step^26^. The “cap-snatching” is carried out by the endonuclease activity of viral polymerase^26^. The PA-Nter endonuclease domain (residues 1–197) has not been identified until recent years^11^,^14^,^15^. However, key active sites that may determine PA-Nter endonuclease activity have not been fully understood. In this study we determined the substitutions, including I61T, V63I, I61T/V63I and Mfour, contributed to variations in the PA-Nter endonuclease activity in the absence of the rest of the polymerase complex. Inspection of the crystal structure reveals that PA-Nter is a member of the PD-(D/E)XK superfamily, which utilizes divalent metal ions for nucleic acid cleavage^14^. Crepin *et al*. introduced mutations into the PA-Nter domain that inactivated the endonuclease activity and showed that these mutations might be involved in the positioning of RNA substrates in the endonuclease active site^16^. Likewise, PA-Nter substitutions (I61T, V63I, I61T/V63I and Mfour) may directly or indirectly affect the binding and/or positioning of the divalent metal ions and DNA/RNA substrates, thereby altering the endonuclease activity. Further studies are required to clarify this issue. Additionally, the PA-Nter protein with the Mfour substitution showed the highest endonuclease activity among all the mutants in the endonuclease assay, yet the substitution did not contribute to the highest virus growth capacity and pathogenicity *in vitro* and *in vivo*. This observation can be reconciled with the potential structural changes of the expressed PA-Nter domain, the involvement of the rest of the polymerase complex in viral transcription and replication, or the direct or indirect interactions of these substitutions with specific host cellular factors^40^. Overall, our data add novel insights into the understanding on the PA-Nter endonuclease domain.

Moreover, the flexible loop region of PA-Nter (residues 53–73) is typically in a disordered structure and its function has been poorly understood^41^. In this study, we demonstrated that the V63I substitution at the flexible loop region of PA-Nter significantly increased the polymerase activity and the endonuclease activity whereas I61T posed a slightly decreased effect. Remarkably, all V63I-containing substitutions that we investigated in this study conferred higher polymerase activity and endonuclease activity. In this case, V63I may act as a dominant substitution when co-exist with other suppressive substitutions including I61T and A37S/V100A. As PA-Nter has been demonstrated to be multifunctional in viral transcription and replication, including endonuclease activity, protein stability, cap binding and virus RNA promoter binding^11^, the V63I substitution may enhance the polymerase activity through interacting with PB1, virus cRNA or vRNA promoters, or the host cellular factors. Desmet *et al*. demonstrated that residues at the PA-Nter flexible loop region suppressed cellular protein synthesis^42^. It is possible that the V63I may contribute to the inhibition of host protein synthesis and thereby increasing virus replication and pathogenicity. With undetermined structure of the flexible loop region^41^, how V63I interacts the rest of the polymerase subunits or other factors remain to be studied.

Yamayoshi *et al*. reported that PA-Nter substitutions S37A and A100V slightly enhanced the virulence of avian H7N9 IAV in mice, though the differences were not statistically significant^25^. In the present study, we further demonstrated PA-Nter combined substitution, A37S/V100A, substantially attenuated virus growth and transcription/replication *in vitro* through decreasing the polymerase activity in avian H7N7 IAV. However, combined with the other two PA-Nter substitutions, the A37S/I61T/V63I/V100A increased the virus pathogenicity *in vitro* and *in vivo*. These findings indicate that the PA-Nter substitution A37S/I61T/V63I/V100A presented by the recently emerged IAVs contributes to an enhanced pathogenicity, though certain residues may pose an attenuated effect.

In conclusion, we demonstrated that the PA-Nter substitutions V63I and Mfour (A37S/I61T/V63I/V100A) increased virus growth capacity and pathogenicity *in vitro* and *in vivo*. The identification of these substitutions as pathogenic and human adaptation determinants may add to the current mutational analysis of the role of PA and aid in the assessment of pandemic potential of avian H7N7 IAV as well as other IAV subtypes carrying these substitutions in humans.

## Methods

### Cells and viruses

Madin-Darby canine kidney (MDCK) cells were grown in Eagle’s minimum essential medium (MEM, Life technologies) supplemented with 10% fetal bovine serum (FBS, life technologies) and 1% penicillin/streptomycin (P/S) at 37 °C with 5% CO_2_. Human embryonic kidney (HEK) 293-T cells were cultured in Dulbecco’s modified Eagle’s medium (DMEM, Life technologies) supplemented with 10% FBS and 1% P/S. Avian influenza A virus (IAV) strain A/Netherlands/219/2003 (H7N7) (WT-H7N7) and mutant viruses were cultured in MDCK cells supplemented with 1% P/S^43^. All virus experiments were conducted in the biosafety level 3 (BSL-3) laboratories^44^.

### Sequence alignment and analyses

Full-length PA protein sequences of various IAV subtypes isolated from human infections were retrieved from the website Global Initiative on Sharing All Influenza Data (GISAID) (http://platform.gisaid.org) (isolate IDs available on request). Multiple sequence alignments were performed using the MEGA 6.06 software. The residues in the PA N-terminal domain (PA-Nter, 1–197 amino acids) were inspected and analyzed among different subtypes to identify the varied ones that might associate with virus pathogenicity.

### Plasmid-based reverse genetics

The eight gene segments of avian IAV strain A/Netherlands/219/2003 (H7N7) were cloned into expression vector pHW2000, respectively^45^. Recombinant viruses were generated by the established reverse genetics^45^. Briefly, virus RNAs of avian IAV strain A/Netherlands/219/2003 (H7N7) were extracted from supernatants of infected MDCK cells using QIAamp Viral RNA Mini Kit (Qiagen). Full-length virus cDNAs were amplified using two-step RT-PCR (primer sequences available upon request). The amplicons were firstly cloned into TA vector using TOPO TA Cloning Kit (Life Technologies) and subsequently transferred into the vector pHW2000. All constructs were confirmed by sequencing.

### Site-directed mutagenesis

PA-Nter substitutions were introduced into plasmid pHW2000-PA (WT-H7N7) using the QuikChange Lightning Site-Directed Mutagenesis Kit (Agilent Technologies).

### Plasmid transfection and virus rescue

The eight plasmids possessing virus gene segments were mixed with ScreenFect A transfection reagent (InCella) according to the manufacture’s protocol and then added into the co-cultured 293-T and MDCK cells in the 12-well plates. The medium was replaced with Opti-MEM (Life technologies) supplemented with 1% P/S at 6 hours post transfection. The transfected cells were cultured at 37 °C for four days to rescue WT-H7N7 and mutant viruses. The viruses rescued were verified by sequencing.

### Virus growth assay

Growth capacity of WT-H7N7 and mutant viruses were investigated. Briefly, the viruses were inoculated into MDCK cells at a multiplicity of infection (MOI) of 1. Cell-free supernatants were harvested at the indicated time points and titrated in MDCK cells by plaque assay^46^.

### Viral mRNA, cRNA and vRNA quantification

A two-step RT-qPCR assay was performed to quantify the abundance of viral mRNA, cRNA and vRNA, which would reflect viral transcription and replication capacity, as reported by Kawakami *et al*.^47^ and Cline *et al*.^48^, with modifications. Briefly, the viruses were inoculated into MDCK cells in 24-well plates at an MOI of 2. The infected cells were collected at 4 and 6 hours post-infection (hpi). Total virus RNAs were extracted using the RNeasy Mini Kit (Qiagen). By using Transcriptor First Strand cDNA Synthesis Kit (Roche), virus cDNAs were synthesized by using primers containing specific genetic tags for viral HA mRNA, HA cRNA and HA vRNA (primer sequences available on request). The cDNA of cellular GAPDH mRNA was synthesized as an internal control.

Real time qPCR was carried out on the LightCycler^®^ 96 (Roche) machine. The primers targeting at the specific tags added in the cDNAs were utilized (primer sequences available on request). Briefly, 5 μl of a 1:10 diluted cDNAs were mixed with 10 μl of 2 × FastStart SYBR Green I Master (Roche), 1 μl of forward primers (10 μM), 1 μl of reverse primers (10 μM) and 3 μl of sterile water. Virus RNA levels were normalized with GAPDH mRNA level^49^.

### Minireplicon assay

The polymerase activity of WT-H7N7 and mutants were investigated as previously described^50^. Briefly, 50 ng each of plasmids pHW2000-PB1, pHW2000-PB2, pHW2000-NP and the indicated pHW2000-PA, together with 50 ng plasmid encoding firefly luciferase reporter flanked by the IAV untranslated region and 100 ng plasmid encoding eGFP, were transfected into 293-T cells cultured in 24-well plates using Lipofectamine^®^ 3000 Transfection Reagent (Invitrogen). The cell lysates were harvested at 24 hours post transfection. Luciferase activity was measured on a Victor X3 multilabel plate reader (Perkin Elmer) by using Bright-Glo™ Luciferase Assay System (Promega). Fluorescence signals were also detected as an internal control for the transfection efficiency. The luciferase readings were normalized with the fluorescence readings.

### Cloning and expression of virus PA-Nter proteins

The DNA fragments of PA-Nter (residues 1–196) were amplified from pHW2000-PA plasmids (with or without the substitutions) and cloned into pET-32a (+) expression vector containing a His_6_-tag (Novagen). The resulted plasmids were then transformed into *E. coli* strain BL21 (DE3) for protein expression^50^,^51^. Briefly, the bacteria were grown to an optical density of ~0.7 at 600 nm (OD600) in LB medium with 100 μg/ml ampicillin at 37 °C. After induction with 0.2 mM isopropyl-β-D-thio-galactopyranoside (IPTG), PA-Nter proteins were expressed by maintaining at 16 °C overnight. The cells were harvested by centrifugation, re-suspended in the lysis buffer (50 mM HEPES, 300 mM NaCl, and 20 mM imidazole, pH 7.8) and sonicated for the release of soluble proteins. After removal of cell debris, PA-Nter proteins were purified using the Ni-NTA Agarose (Qiagen) with the linear gradient of imidazole solutions for washing and elution (the concentration ranged from 20 mM to 250 mM). Unmodified vector was also transformed into *E. coli*, expressed, purified and used as the negative control. Purified proteins were confirmed by 10% SDS-PAGE gel and Western blot^44^. Finally, the proteins were concentrated by using Amicon Ultra-15 Centrifugal Filters (Millipore).

### Fluorescence-based endonuclease assay

Purified PA-Nter proteins were quantified using the Pierce™ BCA Protein Assay Kit (Thermo Scientific). Fluorescence-based endonuclease assay was performed as described previously^50^,^52^. Briefly, 0.2 μg of PA-Nter proteins were incubated with DNA substrates (5′ FAM™-TCTCTAGCAGTGGCGCC-3′BHQ) in a reaction buffer (50 mM HEPES, 150 mM NaCl and 1 mM MnCl_2_, pH 7.8) at 37 °C. Fluorescence signals were released after the cleavage of the DNA substrates by the PA-Nter proteins. The fluorescence signals were measured at the indicated time points on the Victor™ X3 multilabel plate reader (Perkin Elmer).

### Virus infection in mice

Six to eight-week-old female BALB/c mice (11 mice/group) were anesthetized by intraperitoneal injection of ketamine-xylazine (50/5 mg/kg) and intranasally inoculated with 120 plaque-forming units (PFU) of the indicated viruses in a volume of 20 μl. Five BALB/c mice were anesthetized and intranasally inoculated with 20 μl of PBS as a negative control. Mouse body weight and survival were monitored daily for up to 14 days. The mice that lost more than 20% of their initial body weight were humanely euthanized. To evaluate virus replication in mice, 3 mice in each group were euthanized on days 3 and 5 post-infection and mouse lungs were excised. Lung tissues were homogenized in MEM medium and virus titers in the supernatants were titrated in MDCK cells by plaque assay^44^,^53^. All mouse experiments were conducted in accordance with the standard operating procedures of the approved biosafety level 3 animal facilities and were approved by the Committee on the Use of Live Animals in Teaching and Research of the University of Hong Kong.

### Structure analysis

The 3D models of WT-H7N7 and Mfour PA-Nter were prepared by Swiss Model algorithm^54^. Based on the QMEAN score provided by the swiss model server, the best model was selected for further analysis. All the ligand molecules and ions were removed from the PDB files of the models by PDB editor. Furthermore, all the 3D models were structurally validated by SAVES server (http://services.mbi.ucla.edu/SAVES/) with PROCHEK^55^ and the Ramachandran plot was analyzed by using RAMPAGE server^54^. Furthermore, the global sequence alignment was calculated by ALIGN (http://xylian.igh.cnrs.fr/bin/align-guess.cgi) with the template sequences. As PA-Nter proteins retained the endonuclease activity with Mn^2+^ ions^14^, all the models were docked with Mn^2+^ ions, PDB files of which were retrieved from 4E5G (the PDB structure file was retrieved from RCSB data bank) using PDB editor. The PA-Nter models of WT-H7N7 and Mfour were then docked with Mn^2+^ ions at the active sites of receptor by using patchdock algorithm^56^. Afterwards, the protein models in complex with Mn^2+^ ions were docked with 3D model of a single-stranded DNA sequence (5′-TCTCTAGCAGTGGCGCC-3′), which was experimentally proved to be a substrate of the enzyme^52^ and used in our study. The DNA model was built using make-na server (http://structure.usc.edu/make-na/server.html)^57^. The docking was performed using Patchdock/firedock algorthim^56^,^58^. The final docking models after firedock refinement were used to collect ΔG values. Moreover, to evaluate the differences in the PA-Nter proteins of the WT-H7N7 and Mfour, the generated models were compared and aligned with a QMEAN 4, which had the scoring function for the identification of the best model^59^.

### Statistical analysis

Two-tailed Student’s *t*-test, or one-way ANOVA followed with a Dunnett’s test, or Log-rank (Mantel-Cox) test with the GraphPad Prism 6 software package (GraphPad Software, La Jolla California USA) were used for data analysis. *P* < 0.05 was considered significantly different.

## Additional Information

**How to cite this article**: Hu, M. *et al*. Amino acid substitutions V63I or A37S/I61T/V63I/V100A in the PA N-terminal domain increase the virulence of H7N7 influenza A virus. *Sci. Rep.* **6**, 37800; doi: 10.1038/srep37800 (2016).

**Publisher's note:** Springer Nature remains neutral with regard to jurisdictional claims in published maps and institutional affiliations.

## Supplementary Material

Supplementary Information

## Figures and Tables

**Table 1:** The frequency of PA-Nter residues at positions 37, 61, 63 and 100 of influenza A viruses isolated from human infections.

Subtypes	No. of isolates	The frequency of PA-Nter residues							
		37		61		63		100	
		S	A	T	I	I	V	A	V
**H7N9**	400	100%^a^	—b	99.50%	0.50%	100%	—	89.50%	10.50%
H5N6 (in 2015)	1	100%	—	100%	—	100%	—	100%	—
H10N8	3	100%	—	100%	—	100%	—	—	100%
H9N2 (since 2014)	2	100%	—	100%	—	100%	—	—	100%
H7N2	1	—	100%	—	100%	100%	—	—	100%
H7N3	2	—	100%	—	100%	50%	50%	—	100%
**H7N7**	7	—	100%	—	100%	—	100%	—	100%
H5N1	384	—	100%	1.30%	98.44%	0.78%	91.93%	—	87.56%
A(H1N1) pdm2009	4354^c^	0.02%	99.95%	0.03%	99.97%	0.18%	99.82%	5.88%	93.57%
H3N2	7221	—	100%	0.07%	99.65%	0.90%	99.04%	97.31%	2.31%
H1N2	39	—	100%	—	97.44	2.56%	97.44%	79.49%	12.82%
H9N2 (before 2014)	9	—	100%	—	100%	—	100%	—	100%
H6N1	1	—	100%	—	100%	—	100%	—	100%
H5N6 (in 2014)	2	—	100%	—	100%	—	—	—	100%

^a^The percentage represents the frequency of the isolates possessing the indicated residues within each subtype. Full-length PA sequences of the isolates listed above were retrieved from the website Global Initiative on Sharing All Influenza Data (GISAID) (http://platform.gisaid.org) and analyzed in January 2016.

^b^– represents none.

^c^The isolates were those uploaded to the website GISAID in 2009.
