# Supplementary material for: Amino acid substitutions V63I or A37S/I61T/V63I/V100A in the PA N-terminal domain increase the virulence of H7N7 influenza A virus
Source: Sci Rep. 2016 Nov 25;6:37800. doi: 10.1038/srep37800 (PMC5122915; doi:10.1038/srep37800)
Supplement: Supplementary Information [file srep37800-s1.doc]

**Supplementary Information**

**Amino acid substitutions V63I or A37S/I61T/V63I/V100A in the PA N-terminal domain increase the virulence of H7N7 influenza A virus**

Meng Hu^1^, Hin Chu^1^, Ke Zhang^1^, Kailash Singh^2^, Cun Li^1^, Shuofeng Yuan^1^, Billy K. C. Chow^2^, Wenjun Song^1^, Jie Zhou^1*^, Bo-Jian Zheng^1*^

^1^Department of Microbiology, Li Ka Shing Faculty of Medicine, The University of Hong Kong, Hong Kong SAR, China;

^2^School of Biological Sciences, Faculty of Science, The University of Hong Kong, Hong Kong SAR, China.

*Correspondence to:

B.J. Zheng, Tel: +852 22554383, Fax: +852 28551241, Email: bzheng@hkucc.hku.hk; J. Zhou, Tel: +852 22554818, Fax: +852 28551241, Email: [jiezhou@hku.hk](mailto:jiezhou@hku.hk).

Department of Microbiology, The University of Hong Kong, Queen Mary Hospital, 102 Pokfulam Road, Pokfulam, Hong Kong Special Administrative Region, China.

**Supplementary Tables and Figures**

**Supplementary Table S1. The frequency** of PA-Nter residues at positions 37, 61, 63 and 100 of avian H7N7 influenza A virus.

| Positions | Residues | H7N7 influenza A virus | | | |
| --- | --- | --- | --- | --- | --- |
|  |  | Human isolates  (7 isolates) | Mammalian isolates  (13 isolates) | Avian isolates  (75 isolates  since 2013) | Avian isolates  (154 isolates  before 2013) |
| 37 | A | 100%^a^ | 100% | 58.67% | 100% |
|  | S | –^b^ | – | 41.33% | – |
| 61 | I | 100% | 46.15% | 58.67% | 95.45% |
|  | T | – | – | 41.33% | – |
| 63 | V | 100% | 46.15% | 58.67% | 94.81% |
|  | I | – | 53.84% | 41.33% | 1.30% |
| 100 | V | 100% | – | 100% | 98.70% |
|  | A | – | 61.54% | – | – |

^a^ The percentage represents the frequency of isolates bearing the indicated residues among the total isolates within each group. Full-length PA sequences of the isolates listed above were retrieved from the website Global Initiative on Sharing All Influenza Data (GISAID) (http://platform.gisaid.org) and analyzed in January 2016.

^b^ – represents none.

**Supplementary Table S2. The 3D structural differences of PA-Nter models of WT-H7N7 and Mfour.**

| PA-Nter models | Template | Sequence identity | Sequence similarity | Global alignment score | QMEAN4 |
| --- | --- | --- | --- | --- | --- |
| WT-H7N7 | 3hw4 | 98.47 | 0.61 | 1143 | -2.41 |
| Mfour | 3hw6 | 98.47 | 0.61 | 1141 | -5.46 |
|  |  |  |  |  |  |


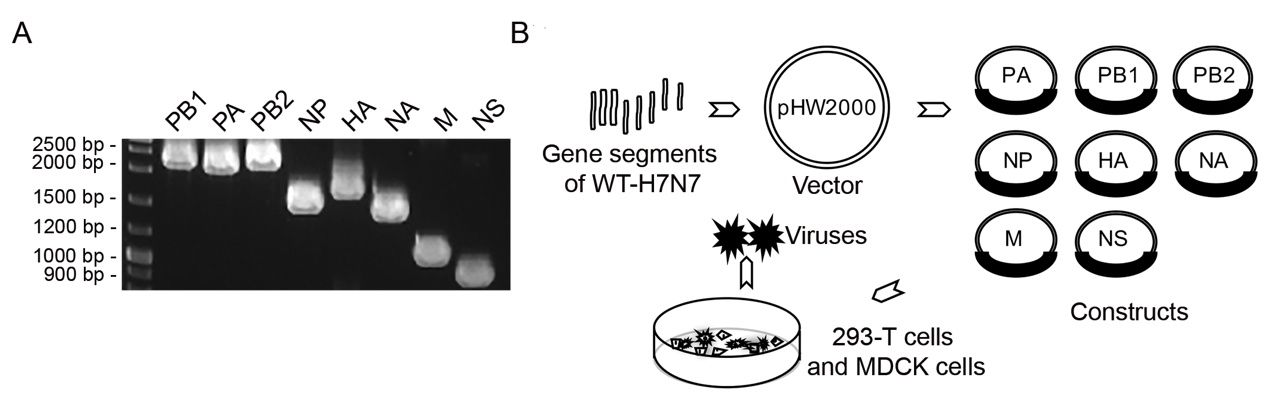


**Supplementary Figure S1. The establishment of reverse genetics of avian H7N7 influenza A Virus (IAV).** (A) Eight DNA segments were amplified from virus genome of avian IAV A/Netherlands/219/2003 (H7N7). (B) Cloning, transfection and virus rescue.


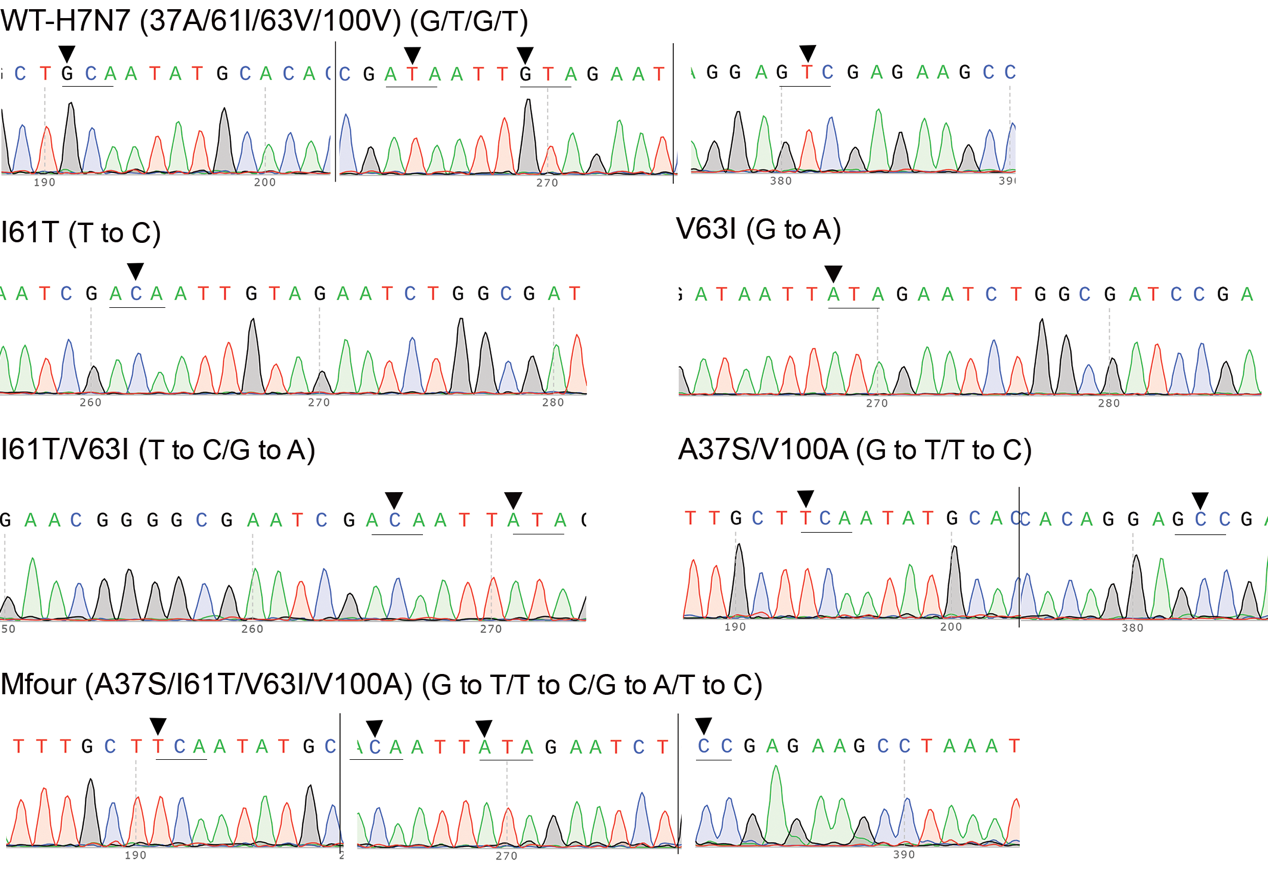


**Supplementary Figure S2. Sequencing results of pHW2000-PA constructs of the WT-H7N7 and the mutants.** The target residues were indicated by triangles and lines.

**
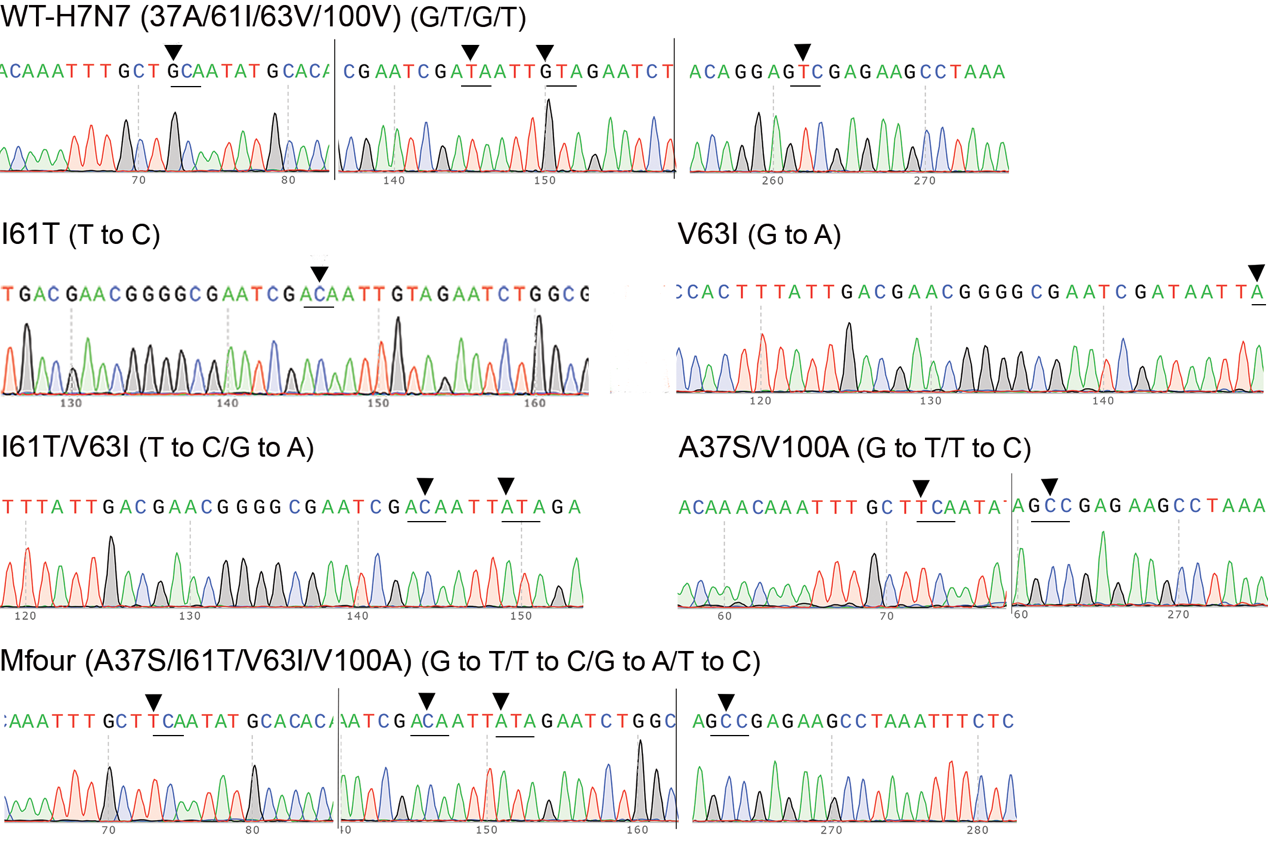
**

**Supplementary Figure S3. Sequencing results of the WT-H7N7 and the mutant viruses.** The target residues were indicated by triangles and lines.

**
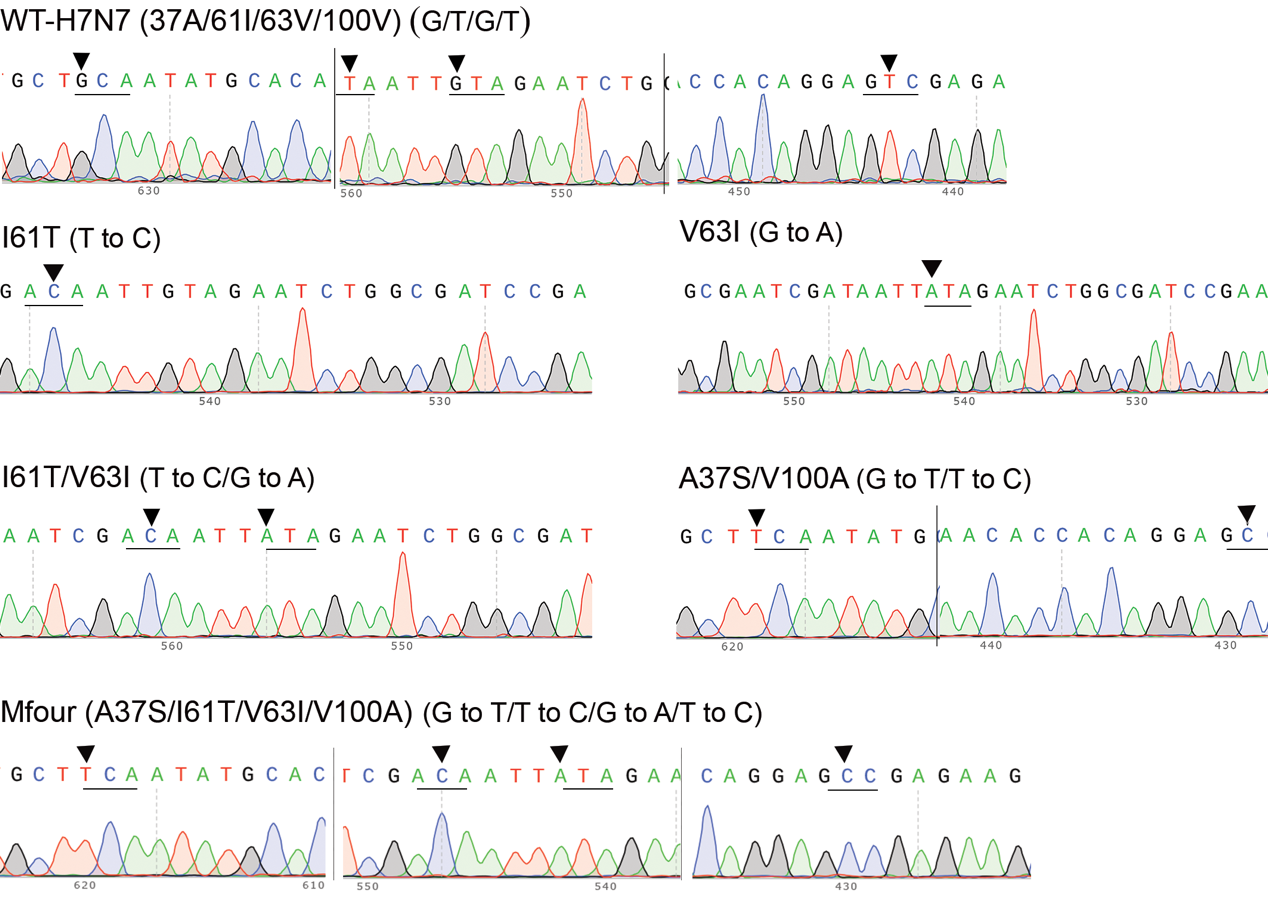
**

**Supplementary Figure S4. Sequencing results of PA-Nter expression constructs of the WT-H7N7 and the mutants.** The target residues were indicated by triangles and lines.
